# Side-to-side esophagogastric anastomosis for minimally invasive Ivor-Lewis esophagectomy: operative technique and short-term outcomes

**DOI:** 10.1007/s13304-021-01054-y

**Published:** 2021-04-26

**Authors:** Manrica Fabbi, Stefano De Pascale, Filippo Ascari, Wanda Luisa Petz, Uberto Fumagalli Romario

**Affiliations:** Department of Digestive Surgery, European Institute of Oncology (IRCCS), 20141 Milan, Italy

**Keywords:** Totally minimally invasive Ivor-Lewis esophagectomy, Esophageal cancer, Intra-thoracic anastomosis, Side-to-side anastomosis, Anastomotic leakage, Complications

## Abstract

Totally minimally invasive Ivor-Lewis esophagectomy (TMIIL) is associated to lower rate of post-operative complication, decreases length of hospital stay and improves quality of life compared to open approach. Nevertheless, adaptation of TMIIL still proceeds at slow pace, mainly due to the difficulty to perform the intra-thoracic anastomosis and heterogeneity of surgical techniques. We present our experience with TMIIL utilizing a stapled side-to-side anastomosis. We retrospectively evaluated 36 patients who underwent a planned TMIIL from January 2017 to September 2020. Esophagogastric anastomoses were performed using a 3-cm linear-stapled side-to-side technique. General features, operative techniques, pathology data and short-term outcomes were analyzed. The median operative time was 365 min (ranging from 240 to 480 min) with a median blood loss of 100 ml (50–1000 ml). The median overall length of stay was 13 (7–64) days and in-hospital mortality rate was 2.8%. Two patients (5.6%) had an anastomotic leak, without need for operative intervention and another patient developed an anastomotic stricture, resolved with a single endoscopic dilation. Chylothorax occurred in three patients; two of these required a surgical intervention. Pulmonary complications occurred in six patients (16.7%). Based on Comprehensive Complications Index (CCI), median values of complications were 27.9 (ranging from 20.9 to 100). The results of our study suggest that TMIIL with a 3-cm linear-stapled anastomosis seems to be safe and effective, with low rates of post-operative anastomotic leak and stricture.

## Introduction

Esophageal resection offers the best chance for cure in patients with locally advanced esophageal cancer. Ivor-Lewis esophagectomy (IL) is the universally accepted technique for disease located in the middle–distal esophagus and gastro-esophageal junction [1]. However, this operation is a complex surgical procedure and associated to higher rates of post-operative complications and morbidities. With the advent of minimally access surgery, IL has rapidly evolved towards totally minimally invasive (laparoscopy and thoracoscopy) approach [2] to minimize surgical trauma and reduce perioperative complications (particularly pulmonary infections), decrease length of hospital-stay and improve quality of life compared to open esophagectomy [3–5]. However, outcomes of minimally invasive esophagectomy (MIE) have been often discordant when considering the incidence of anastomotic leakage (AL) [6–10] probably as a consequence of the technical difficulty of intra-thoracic anastomosis and a long proficiency gain curve for MIE.

Intra-thoracic anastomosis in totally minimally invasive IL (TMIIL) is technically challenging and lacks detailed, generally accepted standardized technique. It is associated with a learning curve, being the refinement of surgical technique an important part of this curve [11, 12]. Individual surgeons starting implementing TMIIL in regular practice refined their technique during implementation, leading to heterogeneous surgical procedures. Thus, a range of options have been described over the years for intra-thoracic anastomosis [2, 13]. Common goal of all techniques is the creation of a safe anastomosis to reduce the risk of leakage and related complications. Nevertheless, there is no accepted ideal approach to perform gastroesophageal anastomosis and search for the optimal procedure is still under study.

In this work, we present our experience with TMIIL utilizing a stapled side-to-side anastomosis in 36 patients with esophageal or esophagogastric junction malignancies. This technique, first reported by Ben-David et al. [14], was modified in our center by introducing the use of a 30 mm linear stapler to perform the anastomosis. The adopted technique was described and discussed in light of the post-operative outcomes.

## Patients and methods

TMIIL was introduced in clinical practice in our department in December 2016 using intra-thoracic side-to-side stapled gastro-esophageal anastomosis. A total of 88 IL were performed from January 2017 to September 2020 (15 open, 34 hybrid with only the abdominal phase performed by a minimally invasive approach and 39 planned totally minimally invasive). All patients had a resectable middle–lower esophageal cancer or Siewert type 1 or 2 esophagogastric junction carcinoma. All patients were initially scheduled for TMIIL except in case of: (i) previous major abdominal or thoracic surgery; (ii) abdominal phase lasting more than 210 min; (iii) bulky tumors (relative contraindication). Of the 39 patients undergoing planned TMIIL, one converted to open approach (side-to-side anastomosis performed during thoracoscopy reinforced in thoracotomy) was included. Instead, in three patients, converted to a hybrid approach (due to, respectively: the transection of tPICC catheter inside the azygous vein at the moment of its division; an unsafe anastomosis; lung injury during trocar insertion due to massive pleural adhesions) a circular end-to-side anastomosis was performed during thoracotomy phase; for this reason, these patients were excluded. Therefore, a total of 36 patients were included in this study and a prospectively collected database was retrospectively reviewed. Data recorded included: demographic characteristics, comorbidities, pre-operative staging, neoadjuvant treatment, intra-operative data, postoperative outcomes and complications, length of hospital stay and mortality, re-admission rate and short-term oncological outcome.

All cases were subjected to a standardized, pre-operative evaluation, discussed in a multidisciplinary setting and indications generally followed international guidelines. Perioperative chemotherapy or neoadjuvant chemoradiotherapy were offered to patients with cT ≥ 3 or node-positive disease. The intervention was performed after 3 weeks and 8 weeks in patients treated with chemotherapy or chemoradiotherapy, respectively. Surgical procedures were performed by a single surgeon skilled in minimally invasive surgery. Pre-operative nutritional jejunostomy was placed during a staging laparoscopy in selected patients candidate to neoadjuvant therapy (for pre-operative nutritional supplementation), or at the time of esophagectomy in the other cases. Hospital stay was calculated from the date of surgery to discharge. Post-operative complications were graded according both to the ECCG and Clavien–Dindo Classification. Comprehensive Complications Index (CCI) was also calculated. Readmission and mortality were recorded for the first 90 days after surgery. The study was approved by Institutional Review Board as does not include any patient identifying information.

### Surgical technique

#### Abdominal phase

**Fig. 1:**
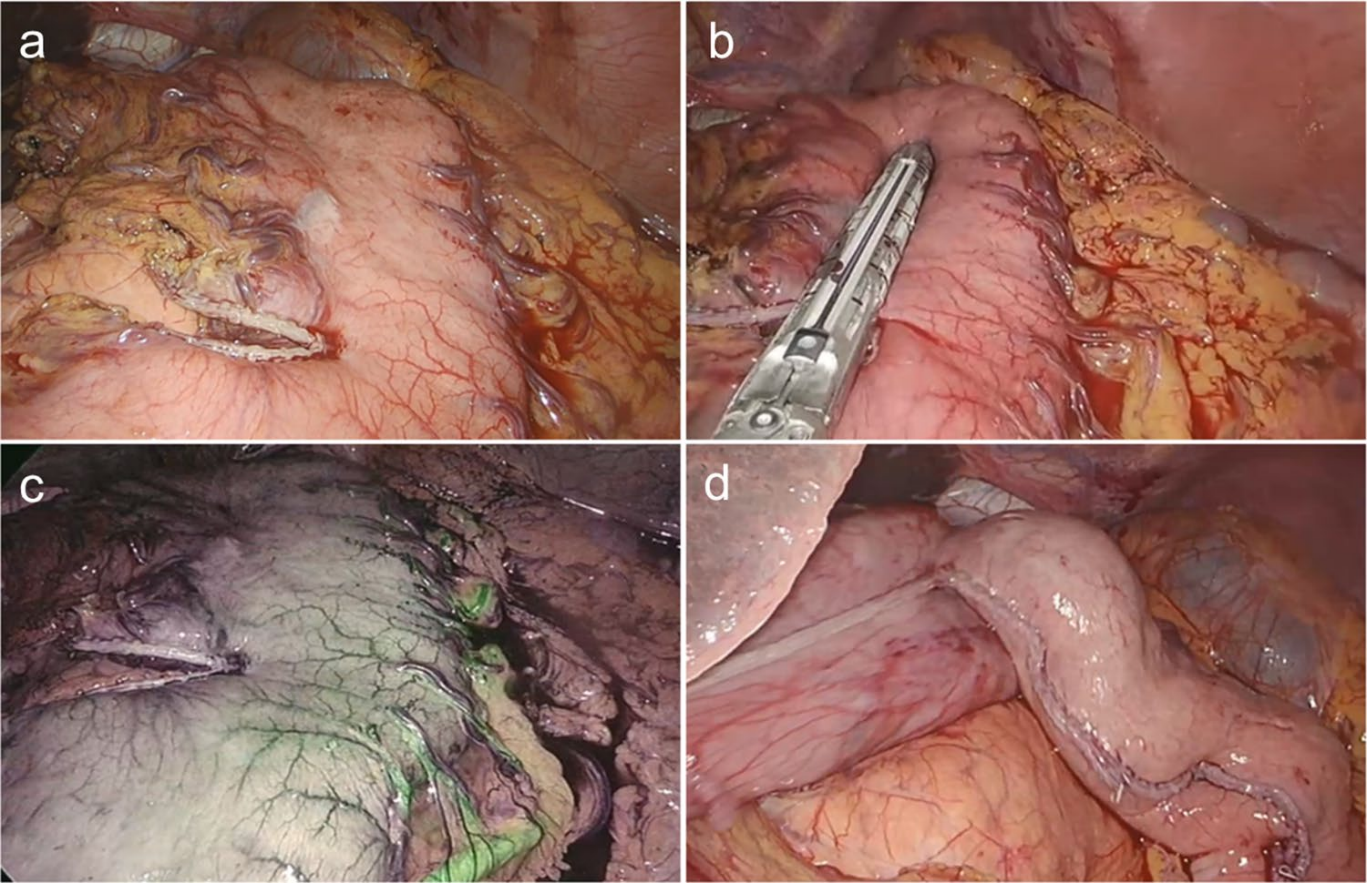
A 4-cm wide gastric tube construction. **a** First cartridge is fired perpendicular to the lesser curve. **b** The others cartridges are fired parallel to the greater curvature. **c** Visualize the gastric blood supply with ICG-fluorescence, after complete gastric mobilization. **d** The bridge at the fundus of the stomach anchoring the specimen to the gastric conduit

The abdominal phase of the operation includes creation of a gastric conduit, lymphadenectomy, and placement of a feeding jejunostomy tube, if not performed previously. With patient in the supine French position, the first 12 mm trocar is placed above the umbilicus (for camera). Capnoperitoneum is established and maintained with a pressure of 12 mmHg. Four additional trocars are inserted: a 12-mm working port in the right mid-abdominal region; three 5 mm ports, in the epigastric site for liver retraction, in the left mid-abdominal region at the midclavicular line epigastric and in the left subcostal region, respectively. The dissection starts by dividing first the gastrohepatic ligament from distally to the crow’s foot, then the phreno-esophageal ligament over the diaphragmatic crus. The crus are dissected free from the gastroesophageal junction. Lymphadenectomy is performed in according to tumor histology, stage and site. The stomach is mobilized by dividing the left gastric vessels, short gastric vessels and gastro-splenic, gastro-phrenic and gastro-colic ligaments (preserving the gastroepiploic arcade). During the intra-abdominal component of the mobilization, the gastric conduit is formed by sequential firings of 45 and 60 mm linear stapler (tri-stapled medium–thick cartridge). The first 45 mm cartridge is fired perpendicular to the lesser curve (distally to the crow’s foot), while the others 45–60 mm cartridges parallel to the greater curvature (Fig. 1a, b). The gastric tube is initiated, but not completed, leaving a bridge at the fundus of the stomach to facilitate the pull-up of the specimen into the chest during the thoracoscopic phase (Fig. 1d). A 4-cm wide gastric conduit is constructed, checking its vascularization with ICG-fluorescence (Fig. 1c). A feeding jejunostomy is routinely placed about 30 cm distal to the ligament of Treitz, secured to the abdominal wall by a self-gripping barbed suture. Pyloroplasty is not performed. At the end of the abdominal phase, an ultrasound injection of ICG in inguinal nodes is performed to visualize the thoracic duct during thoracoscopy; the final number of the injected lymph nodes is operator-dependent (usually two inguinal lymph nodes per side).

#### Thoracic phase

**Fig. 2:**
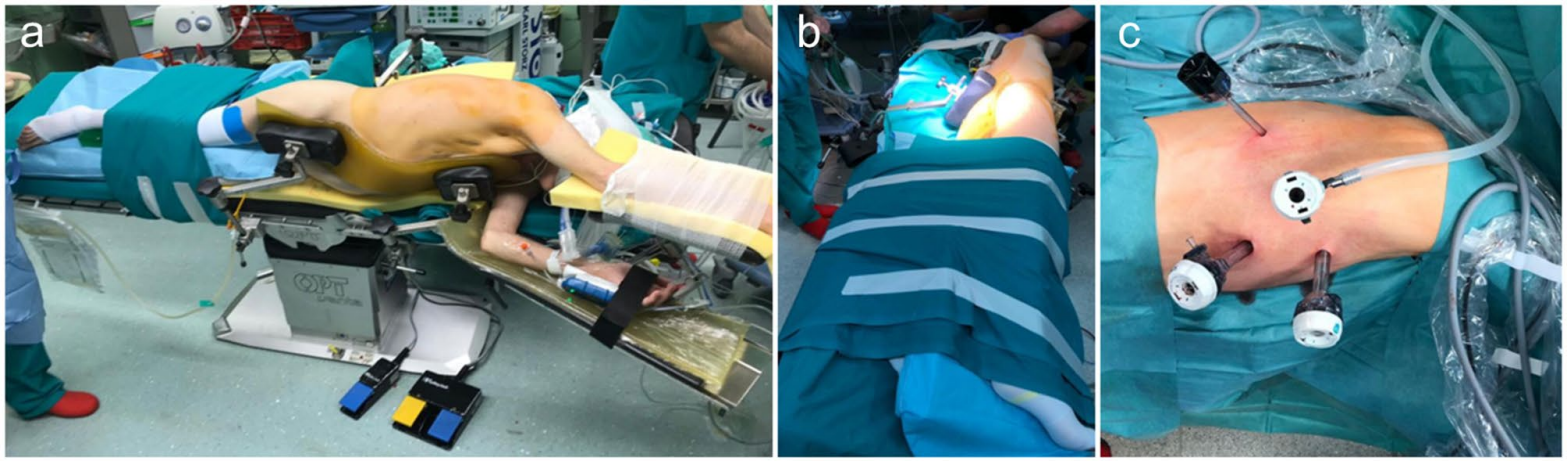
Thoracic phase. **a**, **b** patient’s position: a left lateral decubitus and rotation in a semi-prone position. **c** Trocars position

**Fig. 3:**
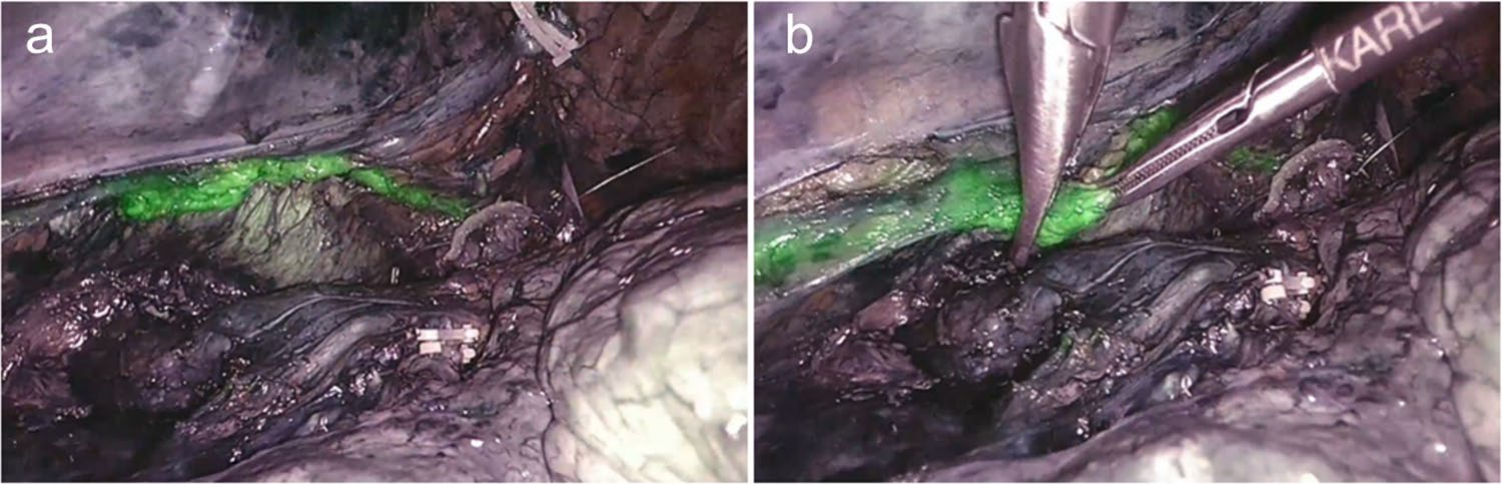
**a** Identification and ICG visualization of thoracic duct. **b** Ligature of thoracic duct with clips

The thoracic phase includes three important steps: esophageal resection, mediastinal lymphadenectomy, and creation of esophagogastrostomy. The patient is positioned in a left lateral decubitus and stabilized on the operative table to allow rotation in a semi-prone position. This position has advantages in terms of visceral exposure and patient ventilation and also allows an expedite switch to thoracotomy if necessary. The right lung is excluded using a left double-lumen tube. Four ports are placed: a 12 mm trocar below the tip of the right scapula, insufflating carbon dioxide until a pressure of 8 mmHg; a 12-mm trocar in the eight intercostal space on the right posterior axillary line; a 12-mm trocar along the middle of the vertebral border of the scapula and a 5-mm trocar in the fifth intercostal space anterior to the scapular tip (Fig. 2). The arch of the azygos vein is divided using hem-o-lock clips (size L) and esophagus is mobilized from above the level of the azygous vein up to the diaphragm (adequate esophageal mobilization is essential to allow the esophagus to overlap 4 to 5 cm onto the stomach). The vagal trunk is usually cut after the emergence of the neural branches for the right bronchus which are preserved. The thoracic duct is preserved if identified under fluorescence imaging; it is selectively ligated if inadvertently or purposely damaged; in case of no visualization, a massive ligation of the azygos vein and the tissue containing the duct is performed (ICG visualization) (Fig. 3). Standard, extended or total mediastinal lymphadenectomy are used according to tumor histology stage and site. The intra-abdominal gastric tube is pulled-up into the thoracic cavity with the staple line facing towards the surgeon as a landmark to prevent rotation of the conduit. The stomach has to be hauled with care through gentle tractions mainly on the omentum and not the gastric walls to avoid injury to the gastric conduit and its vascularization.

#### Anastomotic technique

**Fig. 4:**
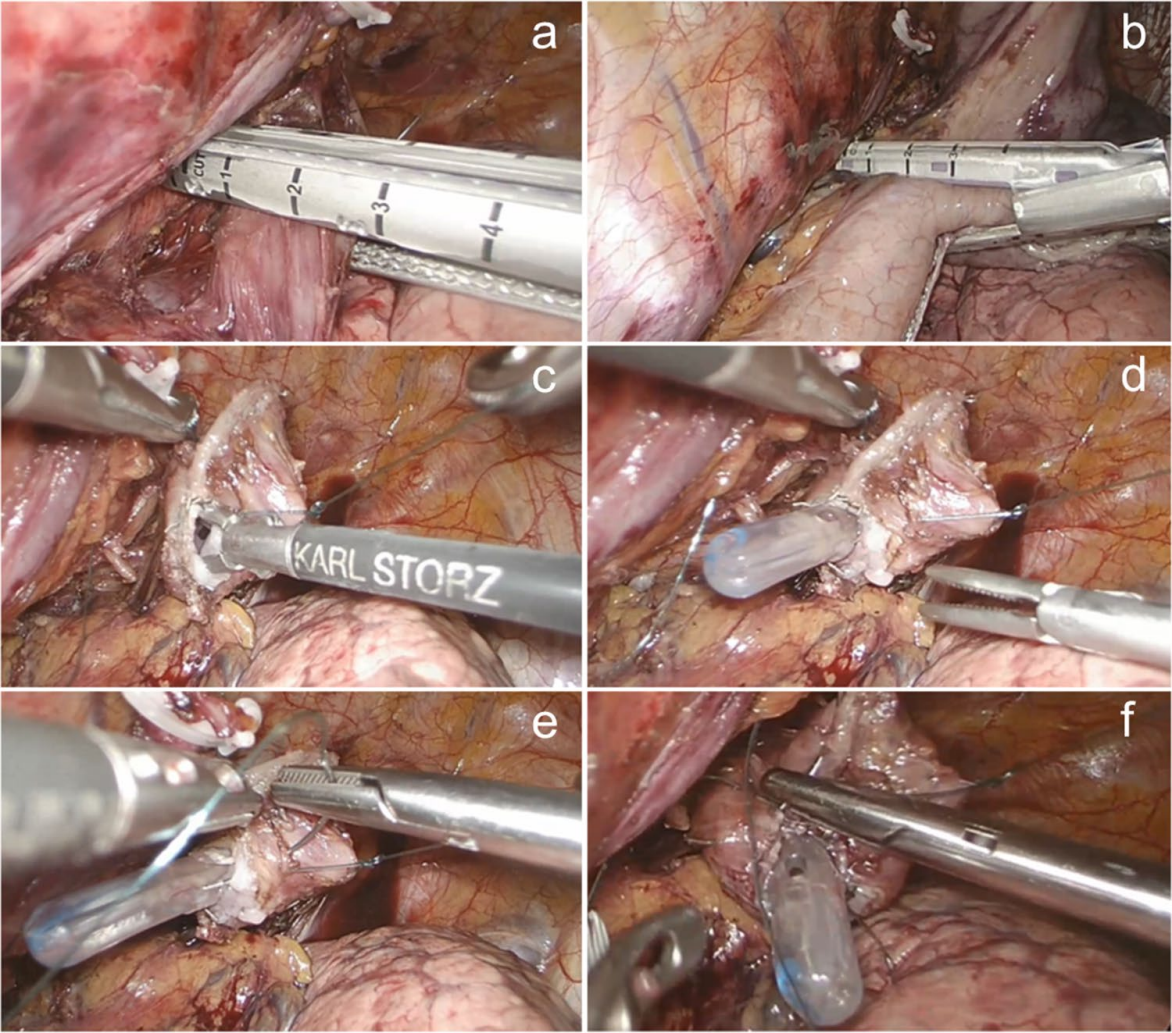
Execution of the esophagogastric anastomosis**.** **a** Transection of the esophagus at the level of the azygous vein with a 45 mm linear stapler (tri-stapled medium–thick cartridge). **b** Completion of the gastric tube by dividing the bridge between the conduit and the specimen. **c** Removing a corner of the staple line on the esophageal stump. **d** The nasogastric tube is pushed through this small esophagotomy to accurately identify the opening. **e**, **f** Placement of two stitches, anteriorly (**e**) and posteriorly (**f**) in the esophageal wall. These stitches transfix all the layers of the esophageal stump wall, to prevent esophageal mucosal retraction

**Fig. 5:**
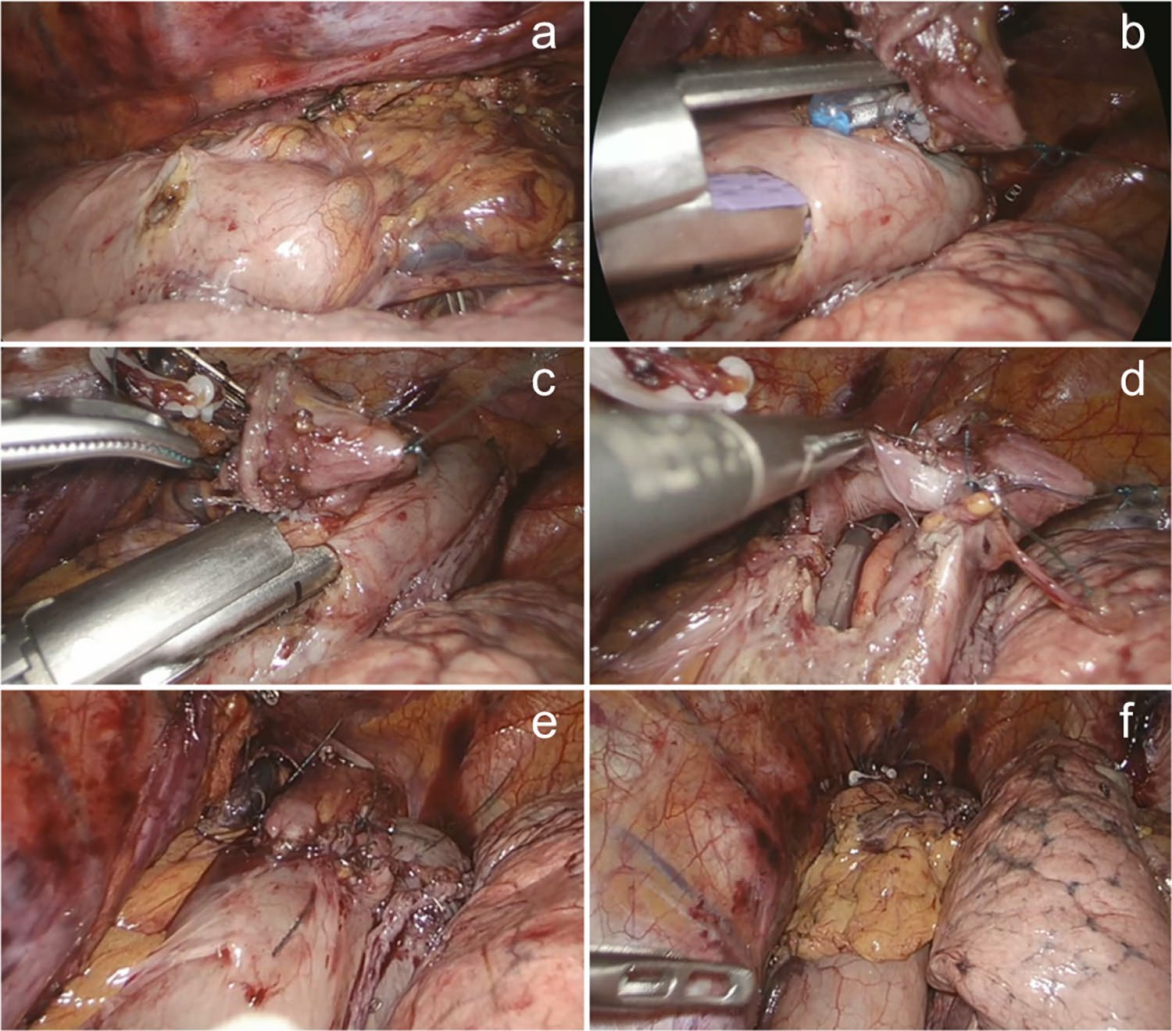
Execution of the esophagogastric anastomosis (continued). **a** A small gastrotomy on the anterior wall of the gastric tube is performed; it is located 5–6 cm away from the top of the conduit. **b** Introduction of a 30-mm linear stapler (tri-stapled medium–thick cartridge) into the esophageal stump and gastric conduit. **c** Removal of the naso-gastric tube and closure of the stapler. **d** Passage of the nasogastric tube in the conduit under direct vision. **e** Closure of the enterotomies by hand-sewn sutures (Maxon® corner stitch and a running self-gripping barbed suture). **f** Omental wrap performed

This phase is illustrated in Figs. 4 and 5. The esophagus is transected where needed above the level of the azygous vein with a 45 mm linear stapler. A corner of this suture is removed using the ultrasonic device, and the nasogastric tube is pushed through this small esophagotomy. Two full-thickness (adventitia to the mucosa) stitches are placed anteriorly and posteriorly in the esophageal wall to prevent esophageal mucosal retraction (technique described by Irino et al. [15]). The gastric tube is completed, by dividing the bridge between the conduit and the specimen (Fig. 4). A small gastrotomy is made on the anterior wall of the gastric tube approximately 5 cm away from the top of the conduit. The esophagogastric side-to-side anastomosis is performed using a 30-mm linear medium–thick cartridge stapler. The enterotomies are closed by hand-sewn sutures using both a Maxon^®^ corner stitch and a running self-gripping barbed suture, after passing a nasogastric in the conduit under direct vision. Care is taken to accurately include the esophageal mucosa in every pass of the suture. A leak test is performed with methylene blue. An omental wrap is performed (Fig. 5). The resection specimen is extracted through a small thoracotomy and the pleural cavity is drained with 28Ch or 32Ch drain.

### Peri-operative protocol

**Fig. 6:**
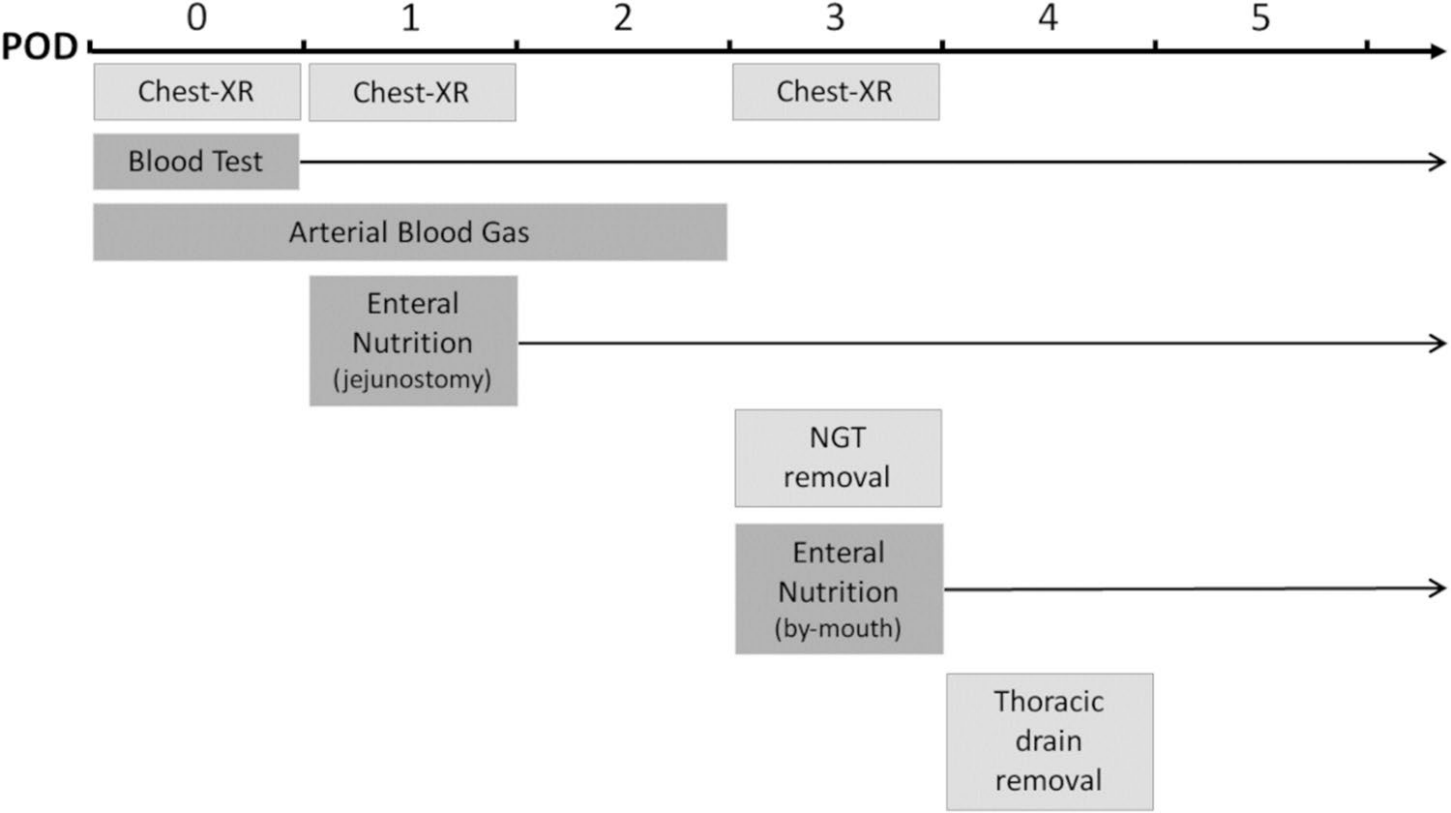
Peri-operative protocol in IEO

An overview of the peri-operative protocol is provided in Fig. 6. The patient is routinely admitted to the intensive care unit for 1 night and, subsequently, transferred to the Upper-GI ward, if no complications occur. Pain is controlled peri-operatively by the use of a thoracic epidural catheter analgesia. Thrombosis prophylaxis (using compression stocking and administration of low molecular weight heparin) is used for the prevention of deep vein thrombosis. Enteral nutrition and water by feeding jejunostomy are started in POD1. Routine blood tests are performed starting from POD0 for monitoring blood inflammatory index at least until POD5. Arterial blood gas is measured until POD2. Routine chest X-ray is done in POD0, 1 and 3. In case of negative chest X-ray (i.e. without indirect signs suggestive of possible anastomotic leak such as new onset pleural effusion, infiltration or air-fluid level in the thoracic cavity), nasogastric tube is removed on POD3 and the patient starts to drink clear liquids. Diet is advanced to a semi-solid food on POD4 and solid diet on POD5. The thoracic drain is removed on POD4 so far as the output is less than 250 ml/24 h and drainage indicative of serous fluid. CT scan, upper GI endoscopy and/or bronchoscopy (bronchial aspiration and BAL) are performed on-demand in case of suspected complications. Patients are typically discharged 7–12 days after the intervention; the home jejunostomy feeding is left if the amount of oral nutritional is not able to cover the entire daily caloric requirement.

## Results

General features of the 36 patients and oncological pre-operative characteristics are reported in Table 1. Most patients were male (69%), with a median age of 65 years (29–83 years). The median body mass index (BMI) was in the healthy range (23 kg/m^2^). The median Charlson Comorbidity Index (CCI) was 4, being arterial hypertension, diabetes mellitus and cardiopathies the most frequent comorbidities. Most esophageal neoplasms were adenocarcinomas (ADC) (80.5%) or squamous cell carcinomas (SCC) (16.7%). Only one patient was affected by primary malignant melanoma of the esophagus. Most of the cases were classified in the clinical stage II and III. Regarding pre-operative therapy, patients with ADC were treated with chemotherapy (10) or chemoradiotherapy (7), while all patients with SCC received chemoradiotherapy (except one psychiatric case). Four patients (11.1%) had undergone a previous endoscopic submucosal dissection (ESD), without criteria for radicality at the final histopathological examination.

**Table 1:** Patients demographic characteristics and pre-operative oncological data

**Parameters (36 patients)**	
Gender M/F (%)	25/11 (69/31)
Age, median (range)	65 (29–83)
ASA, median (range)	2 (1–3)
BMI (Kg/m^2^), median (range)	23 (15.9–31.5)
CCI, median (range)	4 (2–7)
**Comorbidities, number (%)**	
No comorbidities	15 (41.6)
Arterial hypertension	7 (19.4)
DM	1 (2.8)
Arterial hypertension and DM	3 (8.3)
Arterial hypertension and chronic liver disease	2 (5.6)
Myocardial infarction or Congestive heart failure or Atrial fibrillation	6 (16.7)
COPD	2 (5.6)
**Histotype, number (%)**	
Adenocarcinoma	29 (80.5)
Squamous cell carcinoma	6 (16.7)
Other	1 (2.8)
**Pre-operative treatment, number (%)**	
Perioperative chemotherapy	10 (27.8)
Neo-adjuvant chemo-radiotherapy	12 (33.3)
ESD	4 (11.1)
No treatment	10 (27.8)
**Tumor location, number (%)**	
Lower esophagus	13 (36.1)
Esophagogastric junction	23 (63.9)
**Clinical TNM-stage, number (%)**	
Stage I	7 (19.4)
Stage II	11 (30.6)
Stage III	17 (47.2)
Stage IV	1 (2.8)

*ASA* American Society of Anesthesiologists physical status classification, *CCI* Charlson Comorbidity Index, *DM* diabetes mellitus, *COPD* chronic obstructive pulmonary disease, *ESD* endoscopic submucosal dissection

Operative data and histopathological results are shown in Tables 2 and 3, respectively.

**Table 2:** Operative characteristics

**Parameters (36 patients)**	
Duration of intervention in min, median (range)	365 (240–480)
Blood loss in ml, median (range)	100 (50–1000)
**Type of lymphadenectomy (%)**	
Standard dissection	8 (22.2)
Extended mediastinal dissection	28 (77.8)

**Table 3:** Histopathological results

Parameters	
**pTNM-stage, number (%, 14 patients)**	
Stage 0 (pT0N0)	1 (7.1)
Stage 0 (pTisN0)	2 (14.3)
Stage I	4 (28.7)
Stage II	3 (21.4)
Stage III	3 (21.4)
Stage IV	1 (7.1)
**ypTNM-stage, number (%, 22 patients)**	
Stage 0 (pT0N0)	5 (22.7)
Stage 0 (pTisN0)	1 (4.6)
Stage I	2 (9)
Stage II	6 (27.3)
Stage III	7 (31.8)
Stage IV	1 (4.6)
**Lymphonodes harvested (%, 36 patients) **	
*n*., median (range)	24 (7–66)
**Margin status (%, 36 patients)**	
Negative	33 (91.7)
Positive	3 (8.3)
**Treatment effect grade (%, 22 patients)**	
0 (complete)	7 (31.8)
1 (moderate)	3 (13.6)
2 (minimal)	5 (22.7)
3 (poor)	6 (27.3)
Response not graded	1 (4.6)

The median operative time was 365 min (ranging from 240 to 480 min), with a median blood loss of 100 ml. There was no intra-operative mortality. Median number of lymph nodes harvested was 24. Complete R0 resection was achieved in all patients except in 3 (8.3%) (one patient with focal extension of the neoplasm to the proximal resection margin despite the esophageal resection was done under intra-operative esophagoscopic control and the other two patients with the circumferential resection margins (CRM) involved despite preoperative therapy). Among 22 patients who received pre-operative therapy, a complete pathological response was reported in seven patients (31.8%), (five ADC and two SCC), six of them treated by neoadjuvant chemo-radiotherapy.

Short-term outcomes and post-operative complications are shown in Table 4. According to ECCG Classification, two patients (5.6%) had an anastomotic leakage (without subsequent anastomotic stricture), both successfully treated endoscopically with a self-expandable esophageal stent, after 1 week course of Esosponge® treatment.

**Table 4:** Short-term outcomes and post-operative complications

Short term outcomes (n, %, 36 patients)	
Length of hospital stay (in days), median [range]	13 (7–64)
In-hospital mortality, *n*. (%)	1 (2.8)
Readmission within 30 days, *n* (% 35 pts)	4 (11.4)

Complications (*n*, %, 36 patients)		Clavien-Dindo
**Anastomotic complications**		
Anastomotic leakage	2 (5.6)	
Type I	0	
Type II	2	IIIa
Type III	0	
Anastomotic stricture	1 (2.8)	IIIa
**Chyle leak**	5 (13.9)	
Type I	0	
Type II (type A/type B)	3 (3/0)	II
Type III (type A/type B)	2 (1/1)	IIIb
**Pulmonary complications**		
Pneumonia	4 (11.1)	II
Pleural effusion requiring additional drainage procedure	2 (5.6)	IIIa
Acute respiratory distress syndrome	1 (2.8)	V
**Cardiac complications**		
Dysrhythmia atrial requiring treatment	4 (11.1)	II
**Gastrointestinal complications**		
Delayed conduit emptying requiring delaying discharge	2 (5.6)	I/II
**Urologic complications**		
Urinary retention (reinsertion of catheter/delaying discharge)	1 (2.8)	I
**Infection**		
Wound infection requiring opening wound or antibiotics	1 (2.8)	II
Central IV line infection requiring removal or antibiotics	1 (2.8)	IIIa
Intra-thoracic/intra-abdominal abscess	1 (2.8)	IIIa
Other infections requiring antibiotics	7 (19.4)	II
**Others**		
Hemothorax required reintervention	1 (2.8)	IIIb

One patient developed an anastomotic stricture (about 1 month after the operation), resolved with a single endoscopic dilation. No conduit necrosis was recorded.

Abdominal chylous leakage developed in two patients, both treated conservatively; chylothorax occurred in three patients, two of these (of A and B types, respectively) required a surgical intervention. In these two patients, prophylactic duct ligation was performed during esophagectomy: one patient was operated on before the introduction of ICG thoracic duct visualization; whereas, in the other patient only minimal duct fluorescence was visible at surgery. A lesion of duct and a lesion of the cisterna chyli were diagnosed at re-thoracoscopy, respectively. The first was sutured and the second healed after percutaneous embolization of the cisterna.

Pulmonary complications occurred in six patients (16.7%): two of them had a pleural effusion requiring percutaneous drainage while four had a pneumonia requiring antibiotic therapy. Of the two patients with pleural effusion, one also developed acute respiratory distress syndrome and died on POD 64 due to this respiratory complication (after two left thoracotomies for hemothorax).

Cardiac complications, as atrial fibrillation occurred in four patients without heart and renal failure, and resolved after amiodarone administration. Other complications included two cases of delayed conduit emptying and one case of urinary retention, requiring reinsertion of urinary catheter. An antibiotic therapy was required in 27.8% of patients for infection (wound, or central IV line or BAL without pneumonia).

The median overall length of stay was 13 (7–64) days. In-hospital mortality rate was 2.8% (*n* = 1) and re-admission rate was 11.4% (*n* = 4). Causes of readmission were: recurrent episodes of vomiting and dysphagia for delayed gastric-tube emptying, resolved with erythromycin administration and diet modifications (two patients); a recurrent chyloperitoneum requiring percutaneous drainage; general asthenia and iron-deficiency anemia (with stable hemodynamics) resolved with blood transfusions.

Based on Comprehensive Complications Index (CCI), median values of complications were 27.9 (ranging from 20.9 to 100) (Table 5).

**Table 5:** Complications based on CCI index

*N*. patients with complications: 20 (55.6%)	
*n*. patients, (%)	CCI
8 (40)	20.9
1 (5)	24.2
1 (5)	26.2
4 (20)	29.6
3 (15)	33.5
1 (5)	33.7
1 (5)	42.7
1 (5)	100

## Discussion

A retrospective experience on totally minimally-invasive Ivor-Lewis esophagectomy (TMIIL) using side-to-side linear-stapled anastomosis in patients with esophageal malignancies is described in this study.

Open esophagectomy has been long the standard surgical approach, although minimally invasive techniques (MIE) have been gradually gaining favor among surgeons. The appropriateness of oncologic resection and short-term benefits of MIE versus open approach have been supported by robust randomized multi-center trials showing improved QoL, lower rates of pulmonary infection, with no significant differences in margin status, nodal yield, mortality, or survival [3, 16]. At the beginning, McKeown technique was largely used to avoid intra-thoracic anastomosis, although the Ivor-Lewis procedure represents the current indication in case of tumors located in the middle, lower esophagus and esophagogastric junction (S1 and 2), leaving McKeown for cervical and upper thoracic esophageal malignancy [1, 17]. Nevertheless, the adaptation of MIE procedure for intra-thoracic anastomosis proceeds at slow pace among surgeons, mainly due to the difficulty and heterogeneity of surgical techniques for the intra-thoracic anastomosis.

The path toward optimal results faces a steep learning curve, even in case of surgeons already skilled in MIE techniques. Since the initial reports of TMIIL [18, 19], technical execution of intra-thoracic anastomosis has been a major challenge and associated to surgical learning curve. As evidenced by Van Workum et al. [12] in a multicenter retrospective analysis, operative time and incidence of anastomotic leakage represent the key elements of IL MIE-associated learning curve. The length of this curve ranged from 35 to 40 based on operative time and duration of hospital stay [11], although a longer curve (50–119 case) seems to be required to reduce AL rate, as mean incidence decrease from 18.8% to 4.5% after the plateau had been reached, as recently reviewed by Claassen et al. [20]. Thus, a substantial extra number of patients seem to be exposed et al. risk, with possibly devastating sequelae. Also, the choice of anastomotic techniques and subsequent refinements play a pivotal role in establishing a MIE program. Not uncommonly, some surgeons have changed or modified their initial technique until reaching a certain degree of experience and technical confidence with a specific one [21]. Becoming familiar with a surgical technique rather than putative performance differences among techniques seems to be a major determinant of learning curve.

In our case history using a side-to-side linear-stapled technique, a low leakage rate (5.6%) was found. This outcome is comparable with those reported in a literature survey of studies using the same anastomotic technique, ranging from 2.9 to 15.6% (Table 6). Also the median operative time (365 min) is in line with the range reported in other reports, although longer than the 270 min plateau reported by Van Workum et al. [12]. The small cohort size, pre-selection of patients undergoing TMIIL and conversion to hybrid procedure in case of a prolonged abdominal phase (i.e. > 210 min) may bias the reliability of our observed outcomes; however, total case number (close to the lower limit of the learning curve range), leakage rates and operative time collectively indicate the achievement of a certain surgical proficiency in TMIIL in our center. Which of the most common performed esophageal anastomotic techniques (i.e. circular-stapled, linear-stapled, intra-thoracic or Orvil, hand-sewn) has the lowest leakage rates remains controversial, as little consensus exists and varying outcomes are reported from their comparison. A recent meta-analysis found lower anastomotic leak rates with a linear-stapled esophagogastric anastomosis compared to a completely hand-sewn technique [22], although data derived from mixed studies (i.e. IL or McKeown). Analysis of EsoBenchmark database reported lower AL rate in side-to-side linear-stapled (15.6%) and end-to-side purse-string (13.9%) intra-thoracic esophagogastrostomies compared to end-to-side double-stapling anastomosis (23.3%) [36]. Other literature studies comparing the different mechanical approaches suggest no significant differences among stapling techniques for TMIIL (i.e. side-to-side, end-to-side, or end-to-end) [13, 23]. Irrespective of the small sample size, the low incidence of leakage in our study would seem to suggest an apparently shorter learning curve in the side-to-side compared to other techniques. This hypothesis is also supported in other studies, showing similar relationship between leakage incidence and cohort size. However, available data derives from retrospective or prospective single center studies while the question of the best MIE anastomotic technique should be addressed by accruing a randomized control trial.

**Table 6:** Literature survey of studies reported data of side-to-side anastomotic technique using linear stapler in TMIIL

Author	Sample	Duration of surgery in min, median (range)	Blood loss in ml, median (range)	AL (%)	PCs (%)	Chyle leak	Stricture	LoHS (day)
Ben David (2010) [14]	6	360 (300–480)	nr	0	nr	nr	0	nr
Gorestein (2011) [32]	31	nr	nr	3.2	nr	nr	0	nr
Okabe (2012) [33]	26	499 (365–645)	78 (13–210)	3.8	11.5	7.7	0	19 (14–107)
Dong (2015) [34]	8	nr	nr	0	nr	0	0	nr
Irino (2016) [15]	46	408 (210–549)	248 (25–2550)	8.7	4.3	0	2.2	12 (6–96)
Ben David (2016) [35]	60	*	*	1.7	*	*	*	^a^
Schröder (2019) [36]	109	*	*	15.6	*	*	*	^a^
Kukar (2020) [37]	124	463 (403–515)	nr	7.3	37	1.6	5.1	8 (7–11)
Gao (2020) [38]	34	324 (184–480)	157 (50–400)	2.9	8.8	2.9	nr	10 (7–28)

*AL* anastomotic leakage, *PCs* pulmonary complications, *LoHS* length of hospital stay, *nr* not reported

*Mixed IL and McKeown

The occurrence of anastomotic strictures can be relevant, until 18% in a meta-analysis of TMIIL including both end-to-end and side-to-side techniques [1]. In our case, the incidence of anastomotic stenosis was low (2.8%) and comparable to other studies using LS technique (Table 6). However, stricture required endoscopic dilation within the short-term follow-up period. While the contribution of linear-stapled anastomosis to reduce stricture rate compared to hand-sewn has been supported by various studies [22], differences with other mechanical techniques are less clear. A meta-analysis suggested an increased risk of anastomotic strictures using circular compared to linear stapler [30], probably as a consequence of the diameter of the annular stapler [31].

Regarding other post-operative complications, our experience is comparable with those reported in the literature [24, 25]. The incidence of chyle leak (12.8%) is higher compared to other studies using either side-to-side or others techniques (Table 6, [26, 27]) but comparable with recent reports [28, 29] adopting the more stringent definition proposed by ECCG classification. In our case, this complication is probably due to the extended mediastinal lymphadenectomy performed and, thus, not associated to anastomotic technique.

## Conclusions

The results of our study suggest that TMIIL with a 3-cm linear-stapled anastomosis seems to be safe and effective, with favorable outcomes, low rates of post-operative anastomotic leak and stricture.

This technique seems to be easy to learn and perform and it could be a promising technique for beginners training in minimally invasive esophagectomies.
